# Transcervical approach to oropharyngeal synovial sarcoma: a case report

**DOI:** 10.2144/fsoa-2021-0118

**Published:** 2022-11-04

**Authors:** Wisam Algargaz, Hassan M Abushukair, Fareed Barakat, Issa Mohamad

**Affiliations:** 1 Department of Special Surgery, Jordan University of Science & Technology, Irbid, 22110, Jordan; 2 Department of Special Surgery, King Hussein Cancer Center, Amman, 11941, Jordan; 3 Faculty of Medicine, Jordan University of Science & Technology Irbid, 22110, Jordan; 4 Department of Pathology & Laboratory Medicine, King Hussein Cancer Center, Amman, 11941, Jordan; 5 Department of Radiation Oncology, King Hussein Cancer Center, Amman, 11941, Jordan

**Keywords:** head and neck cancer, oropharynx, radiotherapy, synovial sarcoma

## Abstract

**Aim:**

Synovial sarcomas (SS) are malignant tumors rarely arising in the head and neck region. In most of these cases, the tumor arises in the cervical or hypopharyngeal region, and extremely rarely in the oropharynx.

**Case report:**

Herein, we report the case of a 22-year-old male oropharyngeal SS patient presented with breathing difficulty and dysphagia. The management plan included an emergency tracheostomy, followed shortly by transcervical resection of the oropharyngeal sarcoma tumor, the pectoralis major myocutaneous flap was used for pharyngeal reconstruction, followed by adjuvant radiotherapy resulting in more than 5 years disease-free survival.

**Conclusion:**

SS arising in the oropharynx are extremely rare. Transcervical resection coupled with adjuvant radiotherapy warrants enhanced locoregional control in advanced oropharyngeal cases.

## Plain language summary

Synovial sarcomas rarely arise in the head and neck region, specifically in the oropharynx. We report a case of an oropharyngeal synovial sarcoma patient that was presented with breathing difficulty and dysphagia. The patient underwent tracheostomy followed shortly by resection of the oropharyngeal sarcoma tumor, pectoralis major myocutaneous flap was used for pharyngeal reconstruction followed by adjuvant radiotherapy resulting in more than 5 years disease-free survival which confirms the effectiveness of such modalities in permitting locoregional control.

Synovial Sarcoma (SS) is a rare malignant tumor commonly occurring in younger adults that accounts for almost 5–10% of soft tissue sarcomas [1]. One of the most common causes of SS is the chromosomal abnormality T (x; 18) (P11.2; q11.2), which is reported in around 90% of SS patients [2]. Approximately, half of SS patients present with a localized disease at diagnosis [3]. Considering that SS usually occur at the lower limbs, specifically in the thigh area, non synovium-lined sites are rare and scarcely reported in the literature. Head and neck involvement represent only less than 10% of SS [4]. Most of these sarcomas were reported as case reports in the cervical and hypopharyngeal region [5]. Oropharyngeal SS is extremely rare and therefore poorly reported in the literature.

Here, we describe a rare case of surgically treated left oropharyngeal SS being the first reported in the middle east region. Clinical presentation, imaging, surgical approach and histologic features of the tumor are outlined in this report in compliance with the CARE guidelines [6].

## Case report

A previously healthy 22-year-old male, engineering student was referred to our cancer center with progressive breathing difficulties, dysphagia and weight loss. The patient never smoked and has no family history of malignancies. On initial assessment, the patient was found to have a large left oropharyngeal mass that markedly compromised the airway. Once the airway was secured by tracheostomy performed under local anesthesia, a thorough exam of the pharynx revealed a large left oropharyngeal tumor that extended from the left lower pole of the palatine tonsil down to the hypopharynx inferiorly. Biopsy confirmed the diagnosis of SS.

**Figure 1.**
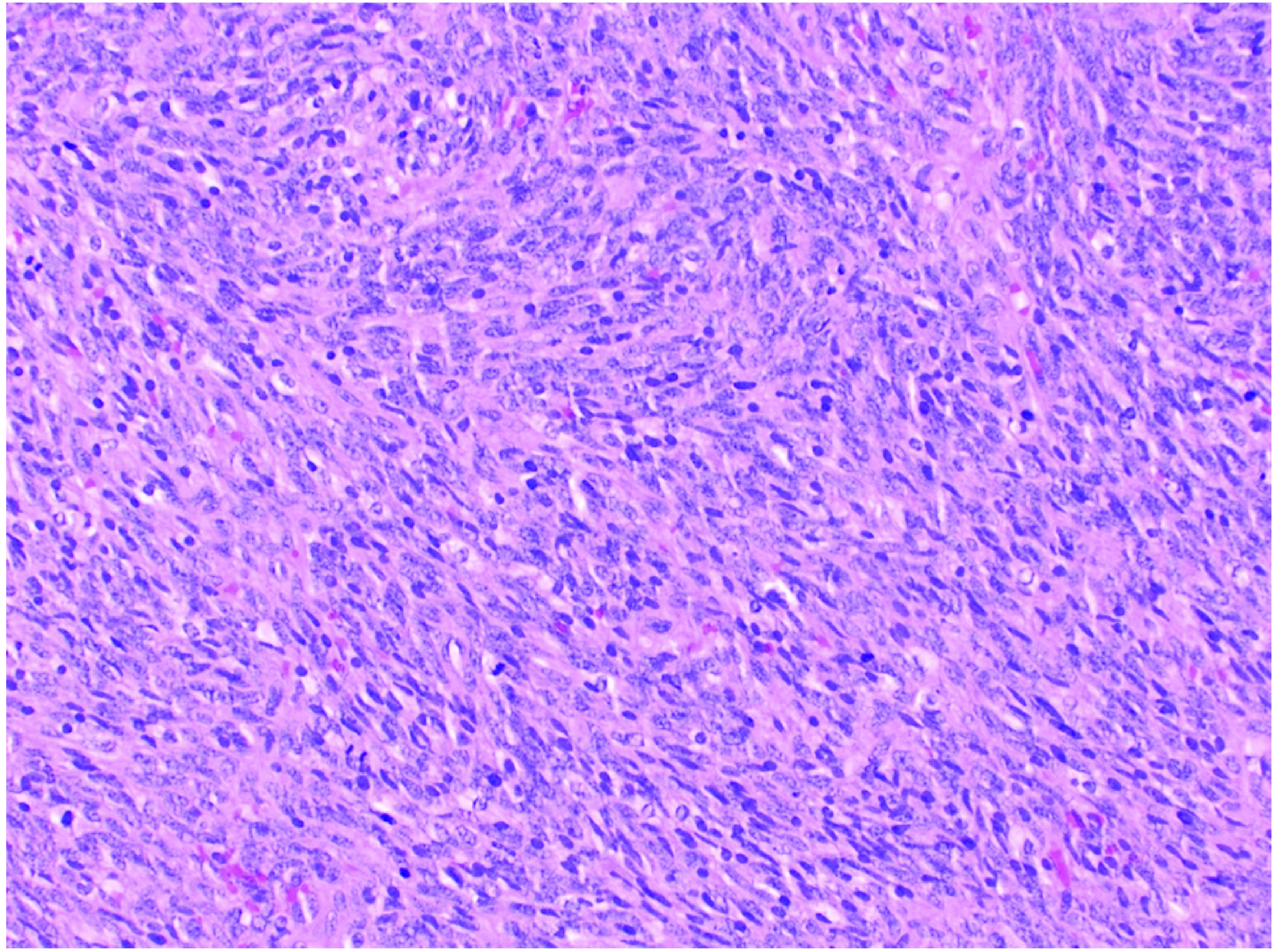
Monophasic spindle cell synovial sarcoma composed mainly of uniform cells with ovoid to spindled nuclei. Tumor cells were positive for the following markers: CD99, BCL-2, TLE-1, CD56 and nestin.

**Figure 2.**
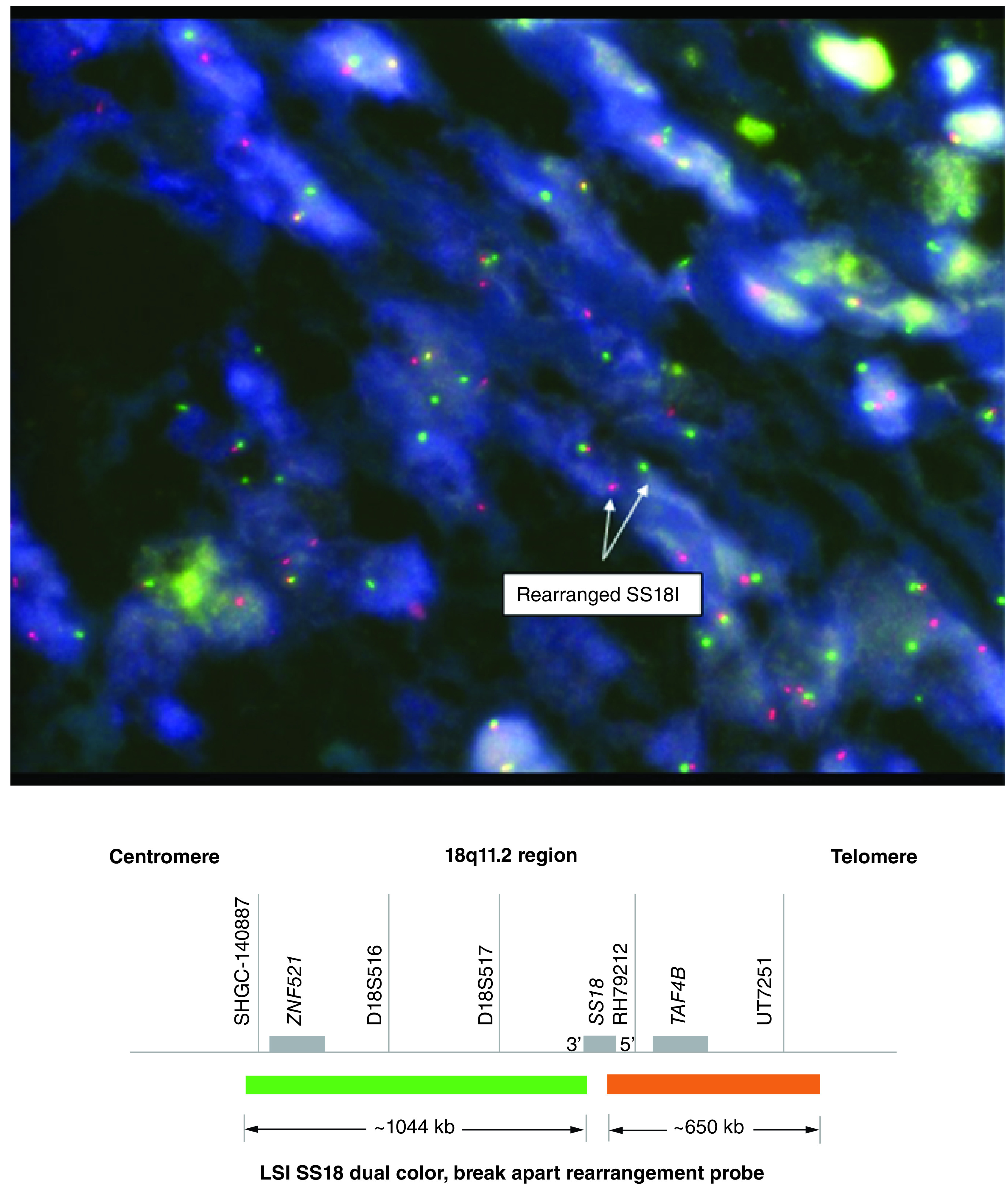
Fluorescence *in situ* hybridization showing SS18 gene rearrangement.

Histologic features included monophasic mesenchymal proliferation composed mainly of uniform spindle cells consistent with SS. Tumor cells were positive for CD99, BCL-2, TLE-1, CD56, Nestin and SS18 rearrangement was identified (Figures 1 & 2). Due to the rarity of this malignancy in the head and neck area, this case was discussed in one of the departmental pathology meetings.

**Figure 3.**
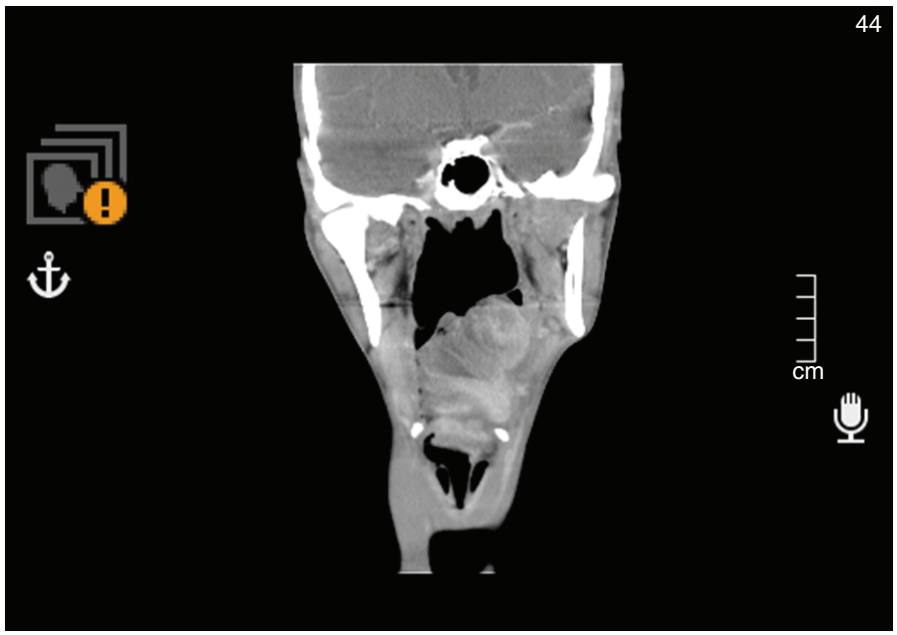
CT scan showing large left oropharyngeal tumor extending from the lower end of the left palatine tonsil to the junction with hypopharynx.

Imaging included neck MRI and PET CT scans. A large left oropharyngeal tumor extending from the lower end of the left palatine tonsil to the junction with the hypopharynx. No regional lymph nodes or distant metastasis were identified, Figure 3.

After discussing the case in our multidisciplinary team meeting, surgical resection was advised. A transcervical approach to the left oropharynx was performed. The procedure involved a transverse cervical incision raising subplatysmal flaps. The intermediate tendon of the digastric muscle was identified and divided. The hypoglossal nerve was mobilized and preserved. Left wide pharyngotomy provided great access to the tumor. This enabled good resection with margins kept under direct vision throughout the procedure. Left palatine tonsil and left half of hyoid bone were included in the resected specimen and found later to be infiltrated by malignant sarcoma cells.

At the end of the tumor resection, it was concluded that the residual pharyngeal mucosa was not sufficient for primary closure and therefore skin in the form of pectoralis major myocutaneous flap was used to reconstruct the defect. Nasogastric tube feeding used for one week until a gastrografin study showed no leak from the pharynx. Oral feeding gradually resumed with the help of the speech and language therapy team. The patient was discharged home on day 14 postoperatively. Our multidisciplinary team recommended adjuvant radiotherapy (RT), as the pathology of the specimen showed a positive margin near the hyoid bone area.

RT was started 5 weeks after surgery. The intensity modulated RT technique was used to deliver 66 Gy over 33 fractions. The high risk clinical target volume (CTV66) was generated by expanding the resected primary gross tumor volume on fused preoperative staging CT scan with a 15 mm margin in superior-inferior direction and 10 mm margin in all other directions, with exclusion of any tissues not at risk for microscopic spread (i.e., the air, uninvolved bone such as mandible, spine and larynx). The low risk CTV56 was generated by expanding the resected gross tumor volume on fused preoperative staging CT scan with a 35 mm superior inferior direction and 15 mm margin in all other directions, including surgical flap, scar and postoperative changes and excluding any tissue not at risk for microscopic disease. No elective RT to the neck nodes was administered. To create planning target volumes (PTVs), CTVs were given a uniform set up margin of 5 mm. The PTV66 and PTV56 volumes were 462 and 950cc, respectively. Target volume coverage and dose constraints for organs at risk were achieved. The treatment was guided by daily bone match cone beam CT image guidance and was well tolerated by the patient.

**Figure 4.**
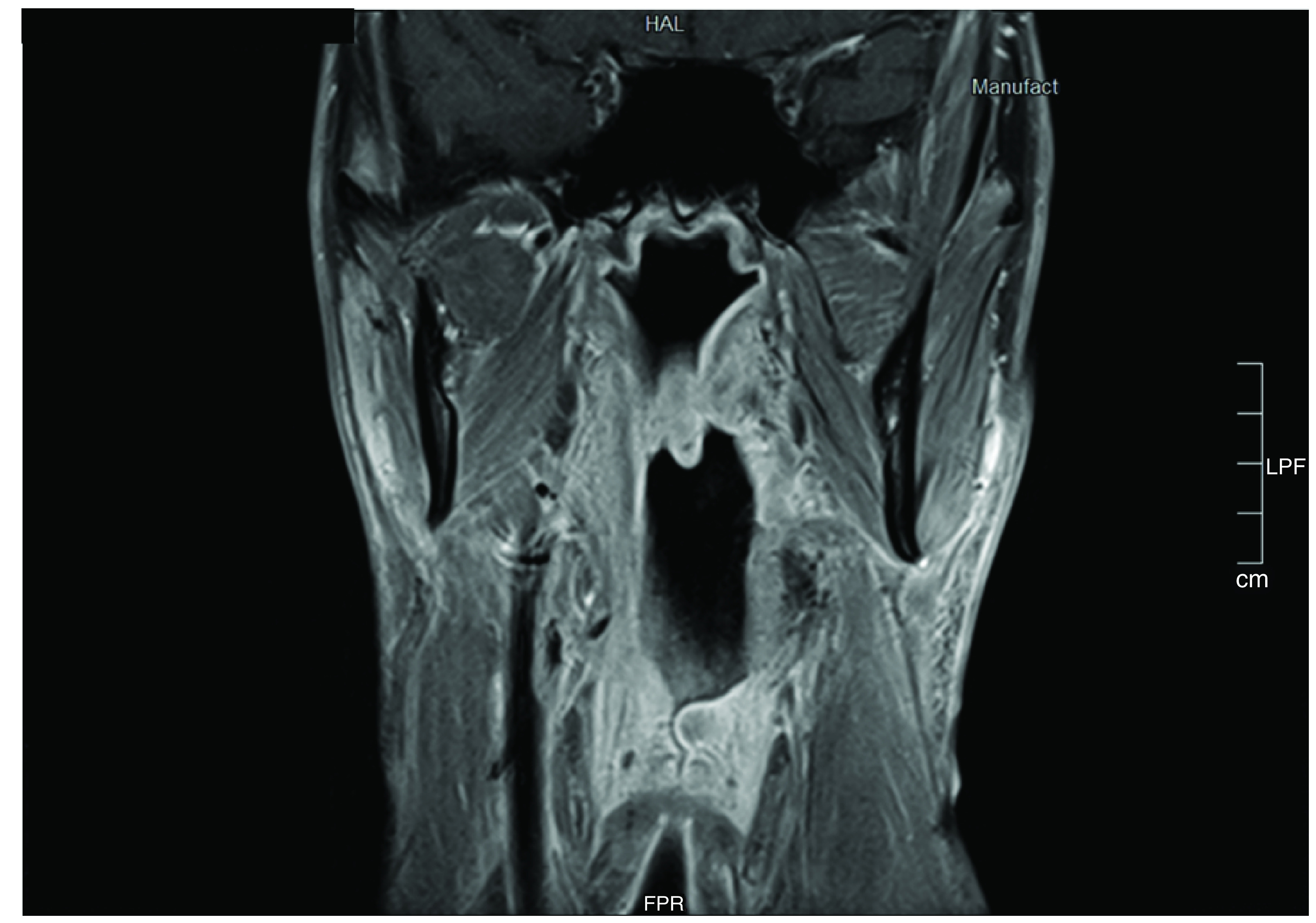
A 5-year postoperative MRI scan of the pharynx.

The tracheostomy tube was removed after the conclusion of RT. Interestingly fiberoptic laryngoscopy has shown metaplasia of the skin used in reconstructing the pharyngeal defect to mucosa like appearance after the first year. Regular follow-up by clinical examination and imaging has not shown any local recurrence or distant metastasis over 5 years of follow-up (Figure 4).

## Discussion

SS is a high-grade aggressive sarcoma that rarely arises in the head and neck region [7]. It is most commonly found in close proximity to large joints, but rarely originates from the joint itself [8]. SS originates from pluripotent mesenchymal cells and comprises around 8–10% of soft tissue sarcomas [9,10].

Pharyngeal SS can be asymptomatic until attaining a large enough size to cause mass effects on surrounding structures leading to serious symptoms like breathing difficulties and dysphagia. In our case, the diagnosis was delayed as the patient only presented after breathing difficulties and dysphagia occurred. CT or MRI remain the primary cross-sectional imaging technique of choice to assess the size of the tumor and regional lymph nodes involvement prior to planning surgical resection. In regards to the histological type, previous studies have shown no significant difference in clinical outcomes between the biphasic and monophasic variants [11].

Adequate surgical resection remains the best treatment option. This can be challenging to achieve in the complex anatomy of the head and neck region. Inadequate transoral wide local excision is followed by high recurrence rates [4]. In this advanced case and after reviewing the images with our radiology colleagues in our multidisciplinary team meeting, we elected to employ an open surgical approach to improve the chance of achieving clear surgical margins. This was in the form of a left lateral transcervical partial pharyngectomy. This provided direct visualization of surgical resection margins. Dissecting and mobilizing the hypoglossal nerve and the hyoid bone improved access to the surgical field.

Head and neck surgeons familiar with the transcervical approach to tongue base tumors will have no difficulty in applying this knowledge to such cases. Other minimally invasive procedures such as the transoral robotic approach have been used and showed favorable outcomes in experienced hands in less advanced tumors [12]. In the absence of such setup, we recommend an open approach as it would be safer and provide better disease control.

The role of radiotherapy with or without chemotherapy remains unclear as previous literature on this topic is scarce. However, some studies have recommended using adjuvant radiotherapy in certain unfavorable pathological features, these include positive margins and larger tumor size as in our case [13]. Although some previous studies have argued the use of chemotherapeutic agents such as doxorubicin for soft tissue sarcomas, we found no solid evidence to support this claim and therefore it was no included in our treatment plan [4,12]. For SS in general, in a national based study from the US, adjuvant chemotherapy with or without radiotherapy was not associated with overall survival in univariate analysis, yet in stage 3 patients both univariate and multivariate analysis showed significant prolongation of overall survival with adjuvant chemotherapy [14]. In another study from the French sarcoma group, neither neoadjuvant or adjuvant chemotherapy had significant correlation with overall survival, progression-free survival, local recurrence-free survival or distant recurrence-free survival [15]. With the emergence of immunotherapy and targeted therapy as viable options that induce durable clinical responses in other solid tumors whether it was in advanced or resectable settings, it is expected that future trials utilizing predictive biomarkers should consider those treatment options in localized SS patients whether it was in adjuvant or neoadjuvant settings. In a phase II trial that administered pembrolizumab, an anti-PD-1, in 10 advanced SS patients, one patient achieved partial response and two achieved a stable disease while the rest progressed [16].

## Conclusion

SS of the oropharynx is extremely rare. Most pharyngeal SS tend to affect the hypopharynx. Only a few cases are reported in the literature describing the oropharyngeal location. Complete surgical resection is the treatment of choice. We found that using the transcervical approach improves the adequacy of the surgical resection and provides better locoregional control. Adjuvant radiotherapy is recommended in advanced cases or in unfavorable pathological outcomes. This treatment plan was effective in our case resulting in 5 years disease-free survival.

**Summary points**

- Synovial sarcomas arising in the oropharynx are extremely rare. The management of such cases is poorly discussed in the literature.
- Complete transcervical resection is the treatment of choice as it provides direct visualization of surgical resection margins enabling for better locoregional control.
- Adjuvant radiotherapy is recommended in advanced cases or in unfavorable pathological outcomes.
